# Gliosarcoma: A Case Series and Comprehensive Literature Review

**DOI:** 10.7759/cureus.103773

**Published:** 2026-02-17

**Authors:** Harvey Misael Aguilar Mora, Laura Karina Barrera Roman, Gervith Reyes Soto, Carlos Castillo-Rangel, Josue Gabriel Agis Ocaña, Manuel Encarnacion Ramirez, Ruben Acosta Garcés

**Affiliations:** 1 Department of Neurosurgery, Hospital Juárez de México, Mexico City, MEX; 2 Department of Oncology, Instituto Nacional de Perinatología, Mexico City, MEX; 3 Department of Neurosurgical Oncology, Mexico National Cancer Institute, Tlalpan, MEX; 4 Department of Neurosurgery, Servicie of the 1ro de Octubre Hospital of the ISSSTE, Mexico City, MEX; 5 Department of Pathology, Hospital Juárez de México, Mexico City, MEX; 6 Department of Neurosurgery, Peoples' Friendship University of Russia (RUDN University), Moscow, RUS

**Keywords:** astrocytic neoplasms, biphasic tumor, central nervous system tumors, gliosarcoma, neurosurgical oncology

## Abstract

Gliosarcoma represents an uncommon primary neoplasm of the central nervous system, distinguished by a biphasic histological architecture that combines glial and mesenchymal elements. In this report, we present three cases of intracranial tumors confirmed histopathologically as gliosarcoma, offering a comprehensive account of their clinical presentation, neuroimaging characteristics, and pathological features. The lesions involved different cerebral regions, specifically the frontal, temporal, and occipital lobes, and manifested with a heterogeneous range of symptoms, including headache, focal motor weakness, and epileptic seizures. The series comprises two male patients and one female patient, aged 54, 51, and 63 years. All patients underwent surgical resection, followed by adjuvant radiotherapy with concurrent and/or adjuvant temozolomide. Despite multimodal treatment, disease progression occurred, consistent with the aggressive biological behavior of gliosarcoma, which is associated with a reported median overall survival of approximately 13-16 months. Through detailed analysis of each case, we highlight the key clinical, radiological, and histopathological findings, emphasizing their diagnostic value and the importance of integrating treatment and outcome data to contextualize prognosis in this rare tumor entity.

## Introduction

Gliosarcoma is a rare and highly aggressive primary neoplasm of the central nervous system, defined by the coexistence of malignant glial and mesenchymal elements within the same tumor. According to the 2021 World Health Organization (WHO) Classification of Tumors of the Central Nervous System, gliosarcoma is currently regarded as a subtype of IDH-wildtype glioblastoma, a designation that reflects shared molecular alterations and similarly aggressive clinical behavior, despite its distinctive morphological appearance [1]. This classification highlights the biological continuum between gliosarcoma and conventional glioblastoma while recognizing the unique histopathological features that distinguish this entity. Although gliosarcoma constitutes only a small fraction of high-grade gliomas, accounting for approximately 2% of all glioblastomas, its clinical impact is substantial and is frequently associated with poor outcomes [2]. Patients typically present with manifestations related to increased intracranial pressure or focal cortical involvement, such as progressive headaches, seizures, and focal neurological deficits secondary to tumor mass effect [3]. From a radiological standpoint, gliosarcoma often poses a diagnostic challenge, as its imaging characteristics may resemble those of other intra-axial high-grade gliomas or even extra-axial tumors, including meningiomas, thereby complicating preoperative assessment [4]. Two principal clinicopathological variants of gliosarcoma have been recognized: primary gliosarcoma, which develops de novo, and secondary gliosarcoma, which arises following treatment of previously diagnosed glioblastoma. Increasing evidence suggests that these forms may differ with respect to underlying molecular alterations, biological behavior, and survival outcomes, with secondary gliosarcoma frequently exhibiting a more complex and treatment-resistant profile [5,6]. However, the rarity of this neoplasm has limited the availability of large, prospective studies, and current knowledge is largely derived from small case series and retrospective analyses, restricting definitive conclusions regarding its natural history and optimal management strategies [7]. In this context, individual case reports continue to play an important role in expanding the understanding of gliosarcoma. Previous reports have emphasized the diagnostic and biological complexity of this entity [8]. Here, we describe three cases of intracranial gliosarcoma, each characterized by distinct clinical presentations and radiological and histopathological findings. The purpose of this case series is to describe the clinical, radiological, and histopathological characteristics of these patients treated at our institution, emphasizing surgical management and clinical evolution in order to illustrate the heterogeneity and aggressive course of this tumor.

## Case presentation

We present three consecutive cases of histologically confirmed gliosarcoma managed at Hospital Juárez de México between 2019 and 2023. All patients were treated by the neurosurgical oncology team at our institution.

Case 1

**Figure 1:**
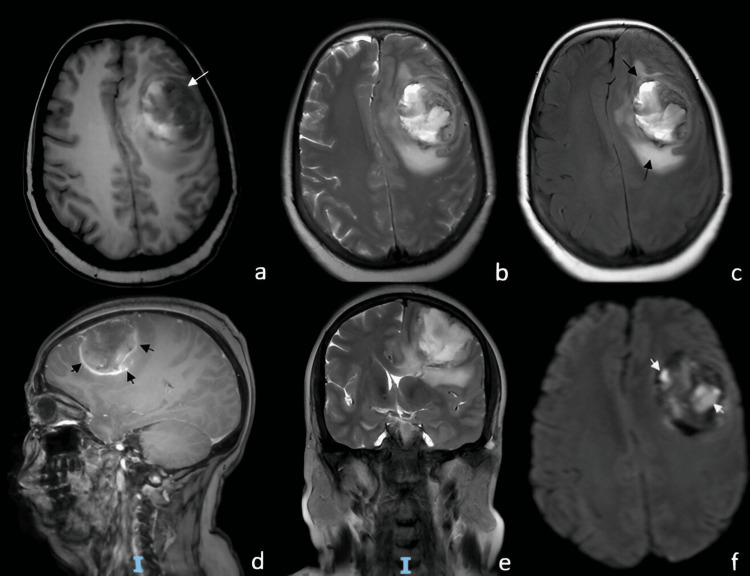
MRI demonstrating a frontal gliosarcoma (a) Axial T1-weighted MRI showing a heterogeneous lesion (long white arrow) involving the left frontal lobe. (b) Axial T2-weighted image illustrating more clearly the mixed-density nature of the lesion, which exerts mass effect on the adjacent brain tissue. (c) Axial FLAIR-weighted image demonstrating significant perilesional edema (long black arrows). (d) Sagittal T1-weighted image with contrast showing predominantly ring enhancement (short black arrows). (e) Coronal T2-weighted image showing marked mass effect with deviation of the frontal horn of the left lateral ventricle and contralateral midline shift. (f) Diffusion-weighted imaging (DWI) revealing apparent diffusion restriction (short white arrows). MRI: Magnetic resonance imaging, FLAIR: Fluid-attenuated inversion recovery, DWI: Diffusion-weighted imaging

**Figure 2:**
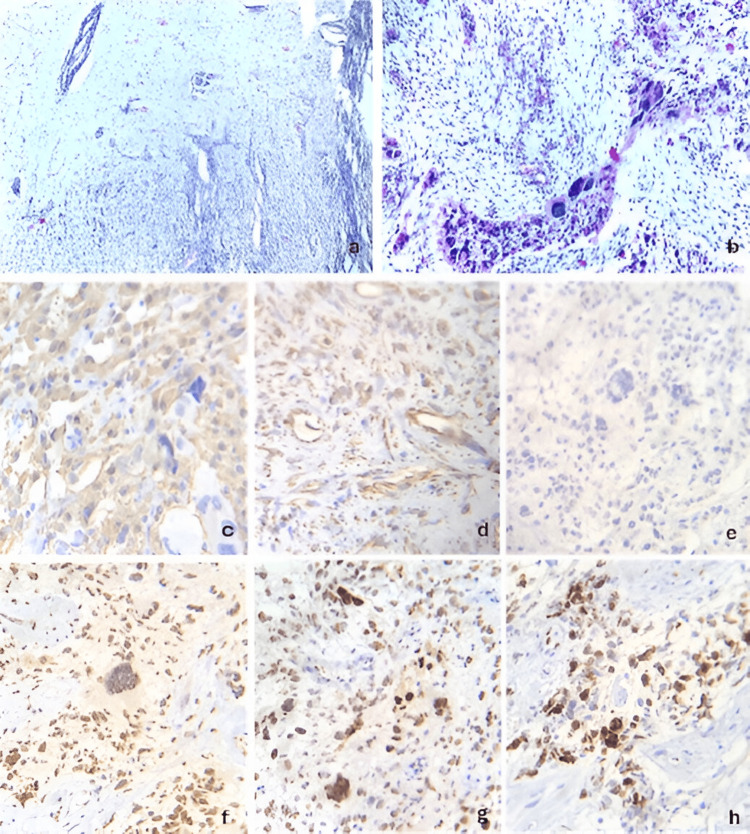
Histopathological and immunohistochemical features of a frontal gliosarcoma (case 1) (a) infiltration of the neoplasm into the cerebral parenchyma (hematoxylin and eosin stain, 4× magnification), (b) pleomorphic neoplastic cells embedded within a loose stroma (H&E stain, 40× magnification), (c) immunohistochemical staining for glial fibrillary acidic protein (GFAP) demonstrating cytoplasmic expression in the glial component and focal expression in the spindle-cell component (40× magnification), (d) vimentin immunostaining showing diffuse membranous expression in both glial and mesenchymal components (40× magnification), (e) IDH1 immunohistochemistry showing absence of expression, consistent with wild-type IDH status (40× magnification), (f) ATRX immunostaining revealing retained nuclear expression, indicating no detectable mutation (40× magnification), (g) p53 immunohistochemistry showing nuclear overexpression (40× magnification), (h) Ki-67 immunostaining indicating a proliferative index of up to 60% (40× magnification). ATRX: Alpha thalassemia/mental retardation syndrome X-linked, GFAP: Glial fibrillary acidic protein, H&E: Hematoxylin and eosin, IDH: Isocitrate dehydrogenase, Ki-67: Cellular proliferation index, p53: Tumor protein p53

A 54-year-old female with a medical history of systemic arterial hypertension and type 2 diabetes mellitus under treatment. Her symptoms began in 2019 with a frontal, oppressive headache of mild to moderate intensity, occurring approximately three days per week. The pain worsened with physical exertion and Valsalva maneuvers and improved with rest and the use of NSAID analgesics. Two weeks later, she experienced generalized tonic-clonic seizures, which remitted after initiating anticonvulsant therapy. Four months afterward, she developed right-sided hemiparesis, predominantly affecting the lower limb, which rendered her unable to walk. On neurological examination, the Glasgow Coma Scale (GCS) score was 15 points; pupils were 2 mm with preserved direct and consensual light reflexes; no papilledema was observed. She presented with a non-dense, disproportionate, and incomplete right brachio-crural hemiparesis. Deep tendon reflexes were hyperactive in the right hemibody, and a pathological right Babinski sign was noted. The following brain magnetic resonance imaging findings are shown in Figure 1. The patient underwent a left frontal craniotomy under general anesthesia. Intraoperatively, a firm, moderately vascularized tumor with areas of necrosis was identified. Although the lesion was located in proximity to functionally critical motor cortex, careful microsurgical dissection allowed gross total resection while preserving neurological function intraoperatively. Early postoperative MRI confirmed no residual enhancing tumor. The histopathological features observed in this case are presented in Figure 2. Postoperatively, the patient received adjuvant treatment according to the Stupp protocol, consisting of fractionated radiotherapy with concomitant temozolomide, followed by adjuvant temozolomide chemotherapy. Despite multimodal therapy, radiological disease progression was documented four months after surgery, corresponding to a progression-free survival (PFS) of four months. The patient died 13 months after diagnosis, resulting in an overall survival (OS) of 13 months.

Case 2

**Figure 3:**
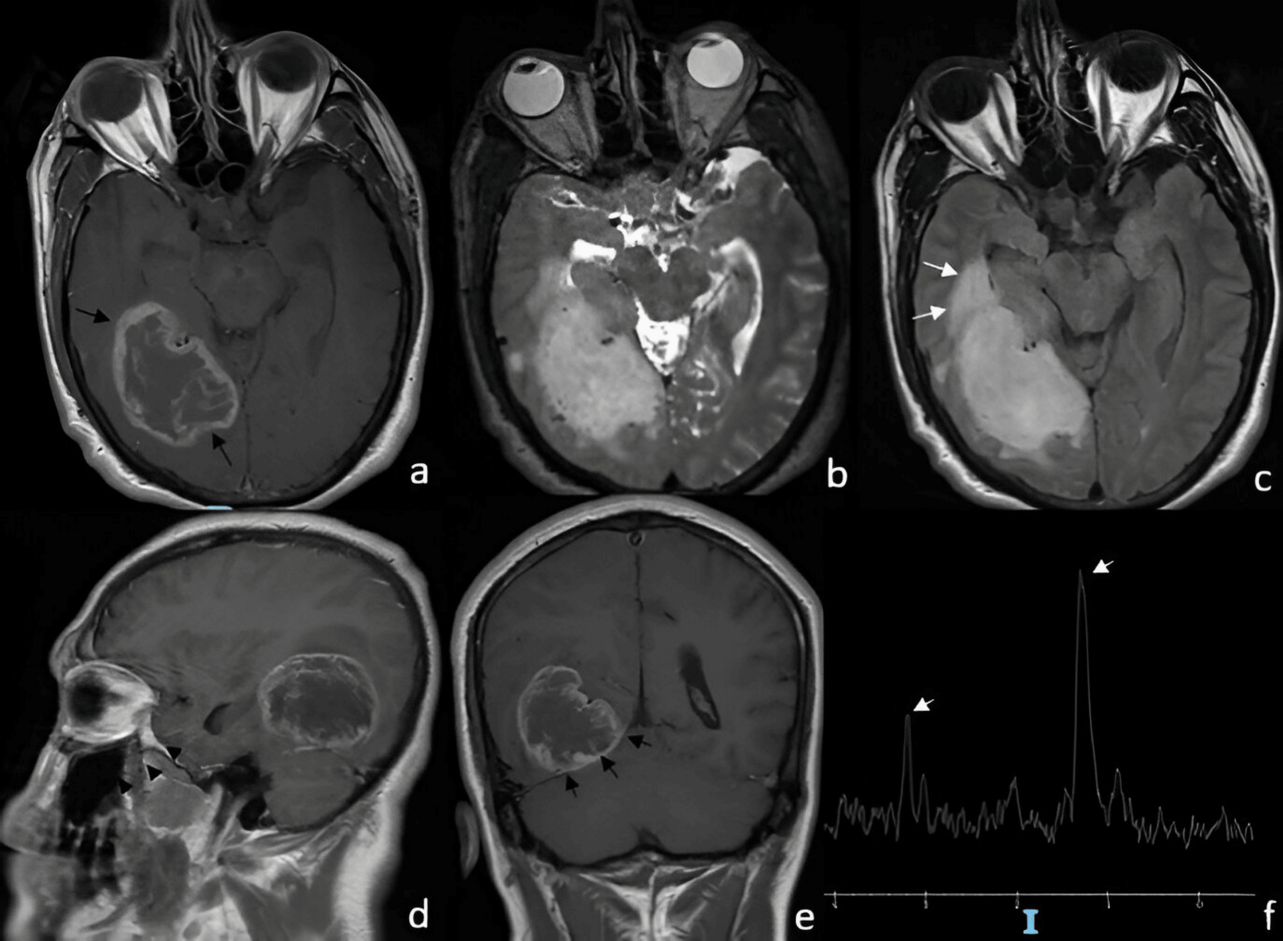
MRI demonstrating a right temporo-occipital gliosarcoma (a) Axial T1-weighted MRI with contrast showing peripheral ring enhancement of the lesion. (b) Sagittal T2-weighted image depicting a homogeneous hyperintense lesion causing mass effect. (c) Axial FLAIR-weighted image showing significant perilesional edema, predominantly in the temporal region (long white arrows). (d) T1-weighted image with contrast revealing the lesion located in the temporo-occipital region. (e) Coronal T1-weighted image with contrast showing a well-demarcated lesion confined to the right supratentorial region. (f) MR spectroscopy showing elevated choline and lactate peaks (short white arrows), along with decreased N-acetyl-aspartate levels. FLAIR: Fluid-attenuated inversion recovery, MRI: Magnetic resonance imaging, MR spectroscopy (MRS): Magnetic resonance spectroscopy, NAA: N-acetyl-aspartate

**Figure 4:**
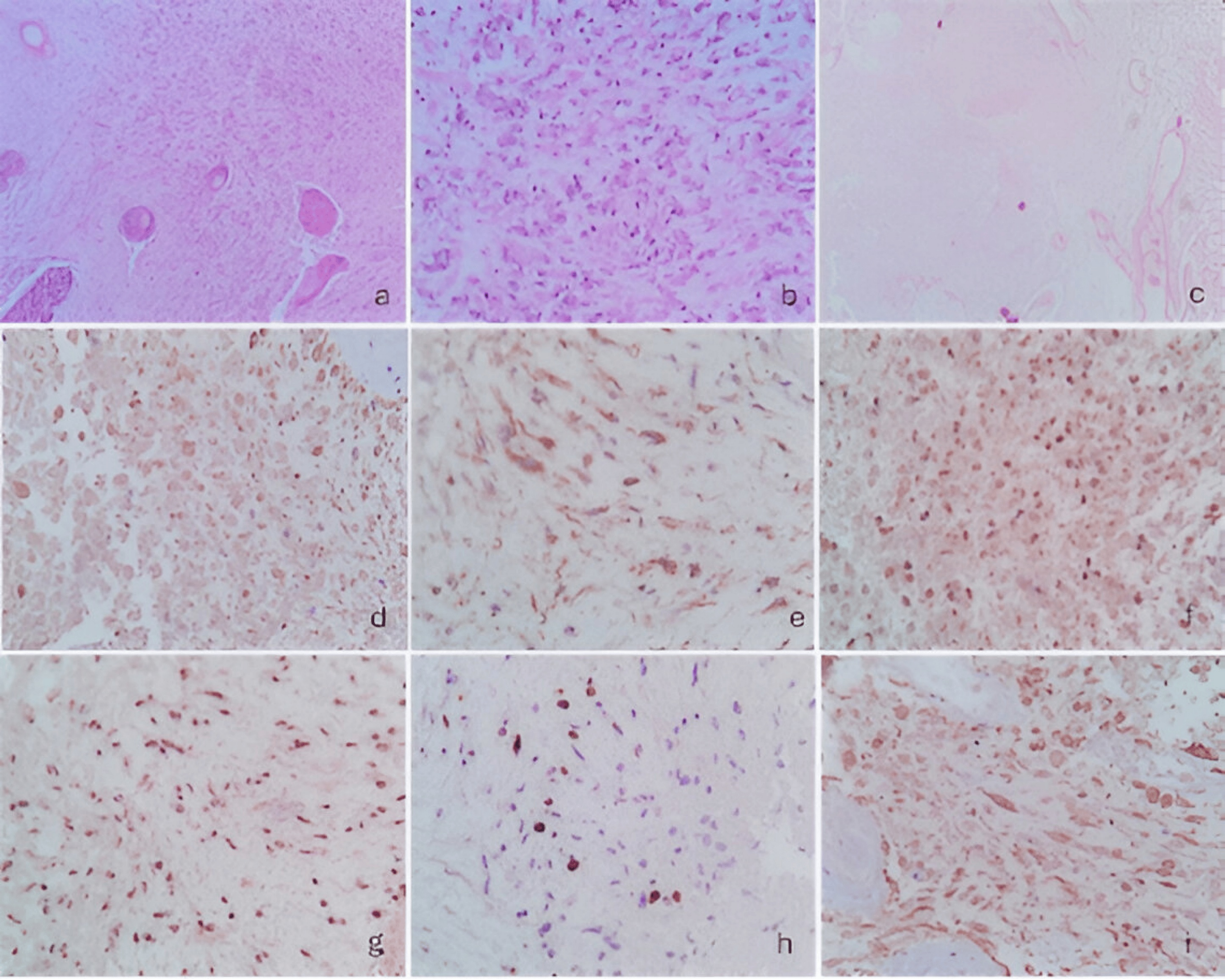
Histopathological and immunohistochemical features of a temporo-occipital gliosarcoma (case 2) (a) Malignant neoplasm showing hypercellularity and a biphasic pattern with spindle-cell and glial-appearing areas, along with marked microvascular proliferation (H&E stain, 10× magnification), (b) Prominent atypia with small vesicular nuclei of astrocytic appearance (H&E stain, 40× magnification), (c) Extensive geographic necrosis (H&E stain, 4× magnification), (d) Positive GFAP immunohistochemical staining in the glial component (40× magnification), (e) IDH1 immunohistochemical staining present in the cytoplasm, indicating wild-type (non-mutated) status (40× magnification), (f) Retained nuclear ATRX expression on immunohistochemical staining (40× magnification), (g) P53 immunohistochemical staining showing nuclear overexpression (40× magnification), (h) Ki-67 immunostaining indicating a proliferative index of up to 50% (40× magnification), (i) and positive vimentin immunohistochemical staining in the sarcomatous component (40× magnification). ATRX: Alpha thalassemia/mental retardation syndrome X-linked, GFAP: Glial fibrillary acidic protein, H&E: Hematoxylin and eosin, IDH1: Isocitrate dehydrogenase 1, Ki-67: Cellular proliferation index, p53: Tumor protein p53

A 51-year-old man with no significant past medical history presented in 2023 with bilateral frontal, pulsatile headaches of moderate to severe intensity. The episodes frequently awakened him from sleep and were accompanied by nausea and vomiting, with only partial relief following the use of NSAID analgesics. Over the following weeks, he reported progressive visual acuity deterioration, more pronounced in the right eye. One month later, he developed mild weakness of the left lower limb, which did not interfere with ambulation. Six months after symptom onset, he experienced status epilepticus characterized by four consecutive generalized tonic-clonic seizures without recovery of consciousness between episodes. Each seizure lasted approximately four minutes, with tonic-clonic activity predominantly involving the upper extremities. He received emergency anticonvulsant therapy, after which his neurological status stabilized. Clinical evaluation revealed a Glasgow Coma Scale score of 15. Visual acuity measured 20/50 in the right eye and 20/30 in the left, and fundoscopy did not demonstrate papilledema. Neurological examination showed a non-dense, proportional, and incomplete left hemiparesis, with hyperreflexia in the left hemibody, presence of patellar clonus, and a left Babinski sign. Figure 3 illustrates the magnetic resonance imaging findings obtained during the diagnostic evaluation. The patient underwent a right temporo-occipital craniotomy under general anesthesia. Intraoperatively, the lesion appeared heterogeneously consistent and moderately vascularized, with relatively well-defined margins superficially. Microsurgical resection was carried out, achieving gross total resection. Postoperative MRI demonstrated complete removal of the enhancing lesion without evidence of residual tumor. Subsequently, the histopathological examination findings are shown in Figure 4. Postoperative MRI demonstrated subtotal resection with minimal residual enhancement adjacent to deep structures. Postoperatively, the patient was treated according to the Stupp protocol, consisting of fractionated radiotherapy with concomitant temozolomide, followed by adjuvant temozolomide chemotherapy. Despite multimodal therapy, radiological disease progression was documented eight months after surgery, corresponding to a progression-free survival (PFS) of eight months. The patient died 14 months after diagnosis, resulting in an overall survival (OS) of 14 months.

Case 3

**Figure 5:**
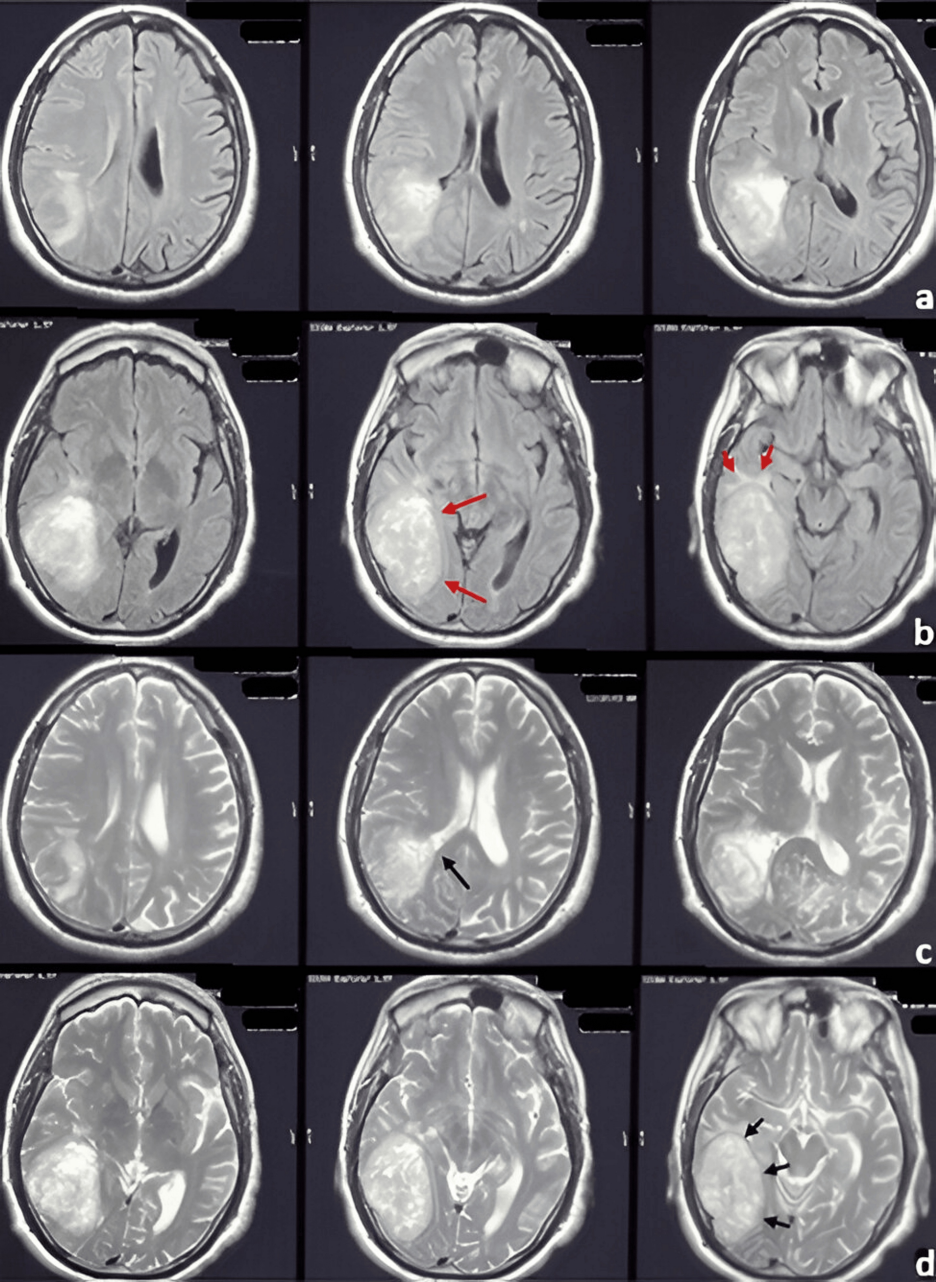
MRI demonstrating a right temporal gliosarcoma (a) Contrast-enhanced T1-weighted MRI showing three axial sections with a right temporal lesion demonstrating heterogeneous enhancement following contrast administration. (b) Contrast-enhanced T1-weighted MRI showing three axial sections with well-defined margins in the medial portion of the lesion (long red arrows) and the presence of predominantly temporal perilesional edema (short red arrows). (c) T2-weighted MRI showing three axial sections demonstrating a close relationship between the lesion and the occipital horn of the right lateral ventricle (long black arrow). (d) T2-weighted MRI showing three axial sections in which a well-defined solid-appearing lesion is observed (short black arrows). MRI: Magnetic resonance imaging

**Figure 6:**
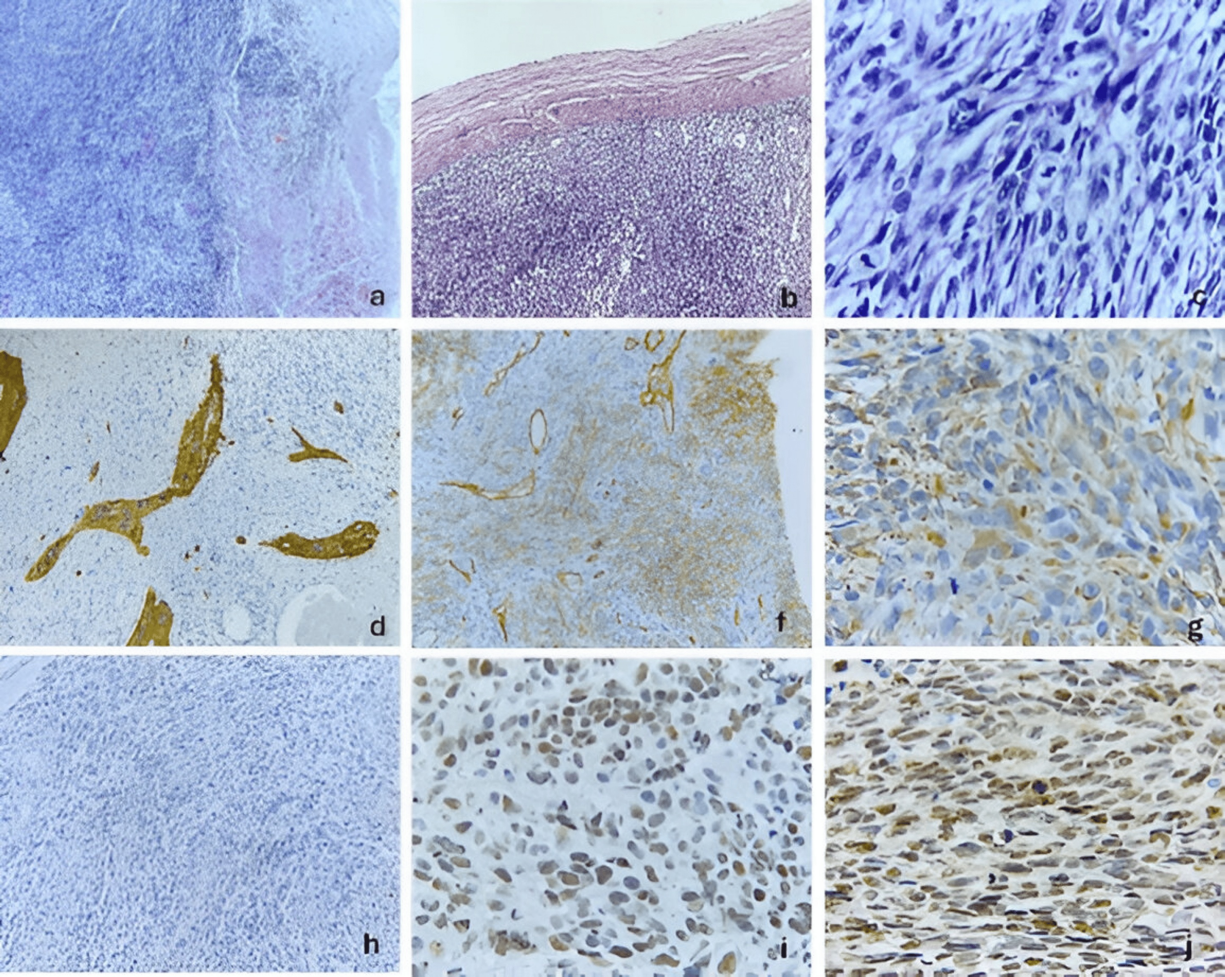
Histopathological and immunohistochemical features of a temporal gliosarcoma (case 3) (a) Neoplasm forming fascicles and whorls, associated with thick-walled blood vessels and tumor necrosis (H&E stain, 4×), (b) Infiltration of the neoplasm into the dura mater (H&E stain, 10×), (c) Spindle-shaped, pleomorphic neoplastic cells with tapered nuclei, coarse chromatin, and numerous atypical mitoses (H&E stain, 40×), (d) Immunohistochemical staining for glial fibrillary acidic protein showing cytoplasmic expression in the glial component (10×), (f) Immunohistochemical staining for CD34 showing membranous expression in the angiosarcomatous component and in the endothelium of associated blood vessels (10×), (g) Immunohistochemical staining for vimentin showing diffuse cytoplasmic expression (40×), (h) Immunohistochemical staining for IDH1 showing absence of expression, indicating wild-type IDH (10×), (i) Immunohistochemical staining for ATRX showing retained nuclear expression, indicating no mutation (40×), (j) Immunohistochemical staining for p53 showing nuclear overexpression (40×). ATRX: Alpha thalassemia/mental retardation syndrome X-linked, CD34: Cluster of differentiation 34, GFAP: Glial fibrillary acidic protein, H&E: Hematoxylin and eosin, IDH1: Isocitrate dehydrogenase 1, p53: Tumor protein p53, Vimentin: Intermediate filament protein associated with mesenchymal differentiation

A 63-year-old man with a history of septoplasty in 2018 following nasal trauma and prostatectomy in 2019 for prostatic hyperplasia began experiencing symptoms in September 2021. He reported right temporal, oppressive headaches of mild to moderate intensity, lasting 60-120 minutes and occurring twice daily. The pain worsened with lifting heavy objects and partially improved with rest and NSAID use. Over the following six months, the frequency of headaches progressively increased. Subsequently, he developed distal-predominant weakness in the left lower limb, although independent ambulation was still preserved. One year after the onset of symptoms, the headache pattern changed, becoming more intense and holocranial, frequently triggered by Valsalva maneuvers and often accompanied by vomiting. Eighteen months after symptom onset, he experienced a focal motor seizure characterized by myoclonic movements of the left upper limb, which later progressed to a generalized tonic-clonic seizure. There was no urinary or fecal incontinence. The seizure lasted approximately three minutes, followed by a 25-minute postictal period and Todd’s paresis affecting the left upper limb. Progressive neurologic deterioration ensued, culminating in left faciobrachiocrural hemiparesis. Neurological examination revealed a Glasgow Coma Scale score of 14, with disorientation in time and space. Pupils measured 2 mm and exhibited preserved direct and consensual light reflexes. A left central facial palsy was noted, along with a non-dense but proportional left faciobrachiocrural hemiparesis. Deep tendon reflexes were hyperactive on the left, and pathological reflexes, including Hoffman, Tromner, and Babinski signs, were present. Subsequently, brain magnetic resonance imaging was performed, as shown in Figure 5. The patient underwent a right temporal craniotomy under general anesthesia. Intraoperatively, the tumor exhibited a solid, fibrous consistency with focal adherence to the dura mater and infiltration into adjacent temporal structures. Due to the involvement of functionally relevant cortical and subcortical regions, maximal safe resection was performed, resulting in subtotal resection. Postoperative MRI demonstrated a residual enhancing tumor adjacent to deep temporal structures. Subsequently, the histopathological features observed in this case are illustrated in Figure 6. Postoperatively, the patient was treated according to the Stupp protocol. Radiological disease progression was documented three months after surgery, corresponding to a progression-free survival (PFS) of three months. The patient died six months after diagnosis, resulting in an overall survival (OS) of six months.

## Discussion

Gliosarcoma (GS) is a World Health Organization (WHO) grade IV tumor and a histological variant of glioblastoma (GBM), characterized by a biphasic architecture with glial and mesenchymal components [1]. Similar to GBM, GS is most frequently IDH-wildtype [2]. Molecularly, alterations commonly observed in primary GBM, such as EGFR amplification and PTEN mutations, are also reported in GS, whereas IDH and TP53 mutations are more characteristic of secondary gliomas. Recent genomic analyses indicate that TERT promoter mutations, along with PTEN and TP53 alterations, represent some of the most frequent somatic events shared by both GBM and GS [3]. Clinically, GS may present de novo or arise secondarily following a prior diagnosis of GBM [4]. Histologically, it retains classical GBM features, including pleomorphic infiltrative glial cells, necrosis, microvascular proliferation, and mitotic activity, alongside areas of sarcomatous differentiation [4,6]. Gene expression profiles in the mesenchymal component, such as SNAI2, TWIST1, MMP2, and MMP9, support a role for epithelial-to-mesenchymal transition in GS pathogenesis [7]. While GS was historically thought to originate from two distinct malignant clones (glial and sarcomatous), current evidence favors a monoclonal origin with mesenchymal transdifferentiation [8]. In the present series, MGMT promoter methylation, TERT promoter mutations, and EGFR amplification were not systematically assessed, which represents a limitation when interpreting treatment response and survival outcomes. As in GBM, the most common presenting signs of GS in both adult and pediatric populations are related to increased intracranial pressure. Seizures are less frequently reported at onset, while symptoms such as aphasia or hemiparesis depend on tumor location. GS occurs predominantly in the supratentorial brain. In pediatric cases, it is most frequently found in the frontal lobes, followed by the parietal and temporal lobes [9]. In adults, GS is also most commonly located in the frontal and temporal regions [10]. Although its exact pathogenesis remains unclear, it is hypothesized that GS originates from a single abnormal cell with specific genetic alterations capable of producing the characteristic biphasic pattern [11]. This hypothesis is supported by the observation of similar genetic changes in both glial and sarcomatous components [12]. Molecular alterations in GS include TP53 and PTEN mutations as well as p16 deletions, which are also observed in both primary and secondary GBMs, although GS exhibits an intermediate incidence between de novo GBM and dedifferentiated low-grade astrocytomas [13]. The genetic pathways of primary and secondary GBMs are entirely distinct [14].

This neoplasm is defined by its biphasic pattern, composed of a glial and a mesenchymal component. Among gliomesenchymal tumors, GS is the most frequent [15]. Two subtypes of GS have been identified, each with distinct prognostic and therapeutic implications: the sarcomatous-predominant subtype, which morphologically resembles a meningioma and shows reticulin production and GFAP negativity; and the gliomatous-predominant subtype, which exhibits necrosis, lacks reticulin, and expresses GFAP [16]. In studies by Han et al. and Salvati et al., the sarcomatous subtype demonstrated better overall survival than the gliomatous subtype. Due to its similarity to meningiomas in location and delineation, sarcomatous GS allows for more feasible radical surgical resection, whereas gliomatous GS tends to arise within the cerebral parenchyma, making complete resection more challenging [12]. Sarcomatous GS with delay of surgery is associated with a poor survival profile, contrasting with the higher response to alkylating agents observed in the gliomatous subtype [10]. This distinction is clinically relevant, as the sarcomatous form shows a higher propensity for hematogenous dissemination, in contrast to local tissue spread and lack of extracranial or cerebrospinal fluid dissemination [14]. Metastases demonstrate histopathologic features exclusively of the sarcomatous component, consistent with classic sarcoma morphology [5]. Histologically, GS is a blend of gliomatous and sarcomatoid tissues, creating its distinctive biphasic appearance. The glial component is of astrocytic lineage and shows all the usual features of GBM, including epithelial differentiation, adenoid gland-like configurations, and squamous metaplasia [17]. The sarcomatous fraction demonstrates nuclear and/or mitotic atypia, with necrosis resulting in a spindle-cell fibrosarcoma-like architecture. Rarely, further mesenchymal differentiation may be observed, including cartilage or bone, smooth or striated muscle, and even a lipomatous component [17]. In the realm of neuroimaging, GS on CT usually presents as a well-defined hyperdense lesion with heterogeneous or ring-like contrast enhancement, reflecting the fibrous sarcomatous component, whereas GBM typically shows low-to-intermediate enhancement [18]. Two imaging patterns have been described: (1) lack of homogeneous enhancement and (2) poorly delineated infiltrative margins [15]. In a study by Zhang et al., GS presented as irregular yet well-circumscribed masses with smooth outer margins resembling meningiomas, regardless of the degree of peritumoral edema [18]. Despite these features, macroscopic findings often lack correlation with radiographic appearance, and several surgical series have reported discordance between imaging and intraoperative findings [18]. GS treatment is multidisciplinary, combining maximal safe surgical resection, radiotherapy, and postoperative chemotherapy, most commonly temozolomide [19]. MGMT promoter methylation status has been shown to predict response to temozolomide in high-grade gliomas, although data specific to GS remain limited. Evidence supporting the use of bevacizumab in GS is scarce and largely extrapolated from glioblastoma series. As in GBM, gross total resection remains the cornerstone of treatment [18]. Several studies have demonstrated a positive correlation between the extent of resection and overall survival. In a clinical study, Zhang et al. showed that extensive resection was associated with improved survival [18]. Regarding radiotherapy, patients receiving postoperative irradiation have significantly longer overall survival compared with those treated with surgery alone [16]. Temozolomide remains the most commonly used adjuvant chemotherapeutic agent, following standard GBM protocols; however, GS response is variable [6]. From a clinical perspective, gliosarcoma should be managed similarly to glioblastoma, with maximal safe resection followed by radiotherapy and temozolomide-based chemotherapy. Molecular profiling, including MGMT promoter methylation, may help inform prognosis and treatment expectations, although GS-specific data remain limited. Evidence supporting second-line or targeted therapies, such as bevacizumab, carmustine, or lomustine, is scarce and largely extrapolated from glioblastoma studies, underscoring the need for dedicated clinical trials in this rare entity [19]. Table 1 summarizes representative published studies on gliosarcoma, including case reports, case series, retrospective and comparative studies, and systematic reviews. Included studies reported original data on adult patients with histologically confirmed gliosarcoma and provided relevant clinical, molecular, or survival information. Gliosarcoma may pose diagnostic challenges due to its radiological overlap with extra-axial tumors such as meningioma. Definitive diagnosis requires histopathological confirmation of the characteristic biphasic pattern, supported by immunohistochemistry demonstrating glial and mesenchymal components. Recognition of sarcomatous versus gliomatous predominance has practical implications, as it influences resectability, dissemination patterns, and treatment response. These cases highlight the importance of integrating imaging, pathology, and clinical context to guide surgical strategy, adjuvant therapy, and prognostic assessment in gliosarcoma.

**Table 1:** Comparative summary of published studies on gliosarcoma Comparative analysis of previously published studies included in the literature review on gliosarcoma.

Author (Year)	Study Type	No. of Cases	Sex Distribution	Main Findings
Gjerdrum & Bojsen-Møller (1999)	Case report	1	1 M	Extraneural metastases with pure sarcomatous component
Parekh et al. (1995)	Case series	17	9 M / 8 F	Median survival 9 months
Perry et al. (1995)	Case series	18	Not specified	Primary vs postradiation gliosarcoma comparison
Salvati et al. (2005)	Case series	11	Not specified	Proposed two histological subtypes
Han et al. (2010)	Case series	20	12 M / 8 F	Median OS 13.9 months; meningioma-like subtype associated with improved survival
Lee et al. (2012)	Comparative study	51 GSM	58.8% male	GSM vs GBM comparison; median OS 13 months
Cachia et al. (2015)	Retrospective study	34	Not specified	PGS vs SGS survival differences
Zhang et al. (2016)	Comparative study	Not specified	Not specified	Survival comparison GS vs GBM
Vuong & Dunn (2022)	Systematic review/meta-analysis	239 PGS / 79 SGS	Not specified	SGS associated with worse OS and PFS

## Conclusions

In the present series, we describe three consecutive patients diagnosed with gliosarcoma at our institution. These cases demonstrate the marked clinical and intraoperative variability of this tumor, ranging from relatively well-circumscribed masses to lesions involving functionally critical regions. Gross total resection was achieved in two patients, whereas one patient required subtotal resection; notably, the latter experienced the shortest overall survival in our cohort. Despite surgical management and adjuvant therapy, early progression was observed in all cases, underscoring the aggressive behavior encountered in clinical practice. Our experience highlights the surgical complexity and biological aggressiveness that characterize gliosarcoma in real-world settings.

## References

[1] Louis DN, Perry A, Wesseling P (2021). The 2021 WHO classification of tumors of the central nervous system: a summary. Neuro Oncol.

[2] Vuong HG, Dunn IF (2022). Primary versus secondary gliosarcoma: a systematic review and meta-analysis. J Neurooncol.

[3] Amer A, Khose S, Alhasan H (2022). Clinical and survival characteristics of primary and secondary gliosarcoma patients. Clin Neurol Neurosurg.

[4] Cachia D, Kamiya-Matsuoka C, Mandel JJ (2015). Primary and secondary gliosarcomas: clinical, molecular and survival characteristics. J Neurooncol.

[5] Lee J, Rodriguez F, Ali SZ (2016). Metastatic gliosarcoma: cytopathologic characteristics with histopathologic correlations. Acta Cytol.

[6] Karsy M, Gelbman M, Shah P (2012). Established and emerging variants of glioblastoma multiforme: review of morphological and molecular features. Folia Neuropathol.

[7] Lee D, Kang SY, Suh YL (2012). Clinicopathologic and genomic features of gliosarcomas. J Neurooncol.

[8] Louis DN, Ohgaki H, Wiestler OD (2017). Biobehavioral survey guidelines for populations at risk for HIV. https://apps.who.int/iris/handle/10665/258924.

[9] Karremann M, Rausche U, Fleischhack G (2010). Clinical and epidemiological characteristics of pediatric gliosarcomas. J Neurooncol.

[10] Han SJ, Yang I, Ahn BJ (2010). Clinical characteristics and outcomes for a modern series of primary gliosarcoma patients. Cancer.

[11] Ohgaki H, Dessen P, Jourde B (2004). Genetic pathways to glioblastoma: a population-based study. Cancer Res.

[12] Salvati M, Caroli E, Raco A (2005). Gliosarcomas: analysis of 11 cases do two subtypes exist?. J Neurooncol.

[13] Perry A, Brat DJ (2010). Practical Surgical Neuropathology: A Diagnostic Approach. Philadelphia: Churchill Livingstone.

[14] Han SJ, Yang I, Tihan T (2010). Primary gliosarcoma: key clinical and pathologic distinctions from glioblastoma with implications as a unique oncologic entity. J Neurooncol.

[15] Romero-Rojas AE, Diaz-Perez JA, Ariza-Serrano LM (2013). Primary gliosarcoma of the brain: radiologic and histopathologic features. Neuroradiol J.

[16] Parekh HC, O'Donovan DG, Sharma RR, Keogh AJ (1995). Primary cerebral gliosarcoma: report of 17 cases. Br J Neurosurg.

[17] Perry JR, Ang LC, Bilbao JM, Muller PJ (1995). Clinicopathologic features of primary and postirradiation cerebral gliosarcoma. Cancer.

[18] Zhang G, Huang S, Zhang J (2016). Clinical outcome of gliosarcoma compared with glioblastoma multiforme: a clinical study in Chinese patients. J Neurooncol.

[19] Palmisciano P, Ferini G, Watanabe G (2022). Gliomas infiltrating the corpus callosum: a systematic review of the literature. Cancers (Basel).

